# Is pass-through of the exchange rate to restaurant and hotel prices asymmetric in the US? Role of monetary policy uncertainty

**DOI:** 10.1186/s40854-022-00425-7

**Published:** 2023-01-11

**Authors:** Uju Violet Alola, Ojonugwa Usman, Andrew Adewale Alola

**Affiliations:** 1 Department of Tourism Guidance, Istanbul Gelisim Universit, Istanbul, Turkey; 2 Economics and Finance Application and Research Center, Department of Economics, Istanbul Ticaret University, Istanbul, Turkey; 3 Centre for Research on Digitalization and Sustainability, Inland Norway University of Applied Sciences, 2418 Elverum, Norway; 4 Faculty of Economics, Administrative and Social Sciences, Nisantasi University, Istanbul, Turkey

**Keywords:** Restaurant and hotel prices, Exchange rate, Monetary policy uncertainty, Energy price index, US economy

## Abstract

This study examines the exchange rate pass-through to the United States (US) restaurant and hotel prices by incorporating the effect of monetary policy uncertainty over the period 2001:M12 to 2019:M01. Using the nonlinear autoregressive distributed lag (NARDL) model, empirical evidence indicates asymmetric  pass-through of exchange rate and monetary policy uncertainty. Moreover, a stronger pass-through effect is observed during depreciation and a negative shock in monetary policy uncertainty, corroborating asymmetric pass-through predictions. Our results further show that a positive shock in energy prices leads to an increase in restaurant and hotel prices. Furthermore, asymmetric causality indicates that a positive shock in the exchange rate causes a positive shock to restaurant and hotel prices. We found feedback causal effects between positive and negative shocks in monetary policy uncertainty and positive and negative shocks in the exchange rate. Additionally, we detected a one-way asymmetric causality, flowing from a positive (negative) shock to a positive (negative) shock in energy prices. Therefore, these findings provide insights for policymakers to achieve low and stable prices in the US restaurant and hotel industry through sound monetary policy formulations.

## Highlights

- The drivers of restaurant and hotel business in tourism destinations are examined.
- There is asymmetric pass-through of exchange rate and monetary policy uncertainty.
- A stronger pass-through is observed during appreciation and a negative shock to monetary policy uncertainty.
- There is asymmetric causality from positive shock in exchange rate to postive shock in restaurant and hotel prices.

## Introduction

The tourism industry is increasingly boosting global economic expansion in a way that industry subsectors (e.g., air travel, medical tourism, restaurant, and hotel) are becoming critical components in many economies. Notably, restaurants and hotels are playing critical roles in the tourism industry’s advancement. Specifically, recent industry trends suggest that restaurants (e.g., coffee shops and fast food sectors) and hotels (for overnight accommodation, e.t.c) are continuously experiencing global growth in market and chain operations (International Labour Organization 2010). Hotels and restaurants may be experiencing continuous global growth owing to the industry’s highly competitive and segmented nature (Statista 2020a, b). Hotels are categorized as either independent or unaffiliated, and restaurants (under the foodservice segment) are categorized as commercial or noncommercial (Statista 2020a, b). In most advanced economies including the United States (US), restaurant and hotel industries are distinctly booming and competitive. Tourists’ purchase intentions significantly determined by destinations’ food being served in the restaurants, hotels’ hospitality and service quality, and other cultural representations (Lee and Choi 2020).

In the US restaurant and hotel industry, annual growth rate in 2023 is expected to be 2.8% higher than 2008 (Statista 2020b). With over 1 million restaurant locations (including global brands and other retail restaurants) and about 15.6 million in jobs, the restaurant industry is expected to amass $899 billion (i.e., expected growth rate of 4% from 2019) in revenue in 2020 (National Restaurant Association 2020). The National Restaurant Association (2020) projects that 1.6 million more new restaurant jobs would be added by 2030, or 17.2 million jobs in total. Sales and jobs created in the restaurant industry would have increased from $590 billion and 12.2 million in 2010, respectively, to $1.2 trillion and 17.2 million in 2030, respectively. Moreover, while the US food service segment comprises more than 80% of the restaurants in the US, noncommercial categories account for about 20%. Moreover, hotel chains account for two-thirds of the restaurant and hotel market. Restaurants and hotels are significant not only to the US economy but also the global tourism industry.

Despite the restaurant and hotel industry’s strong performance, the industry remains vulnerable to various uncertainties, which inhibit the contributions of the industry to global tourism and economic expansion (Akadiri et al. 2019, 2020; Othman et al. 2020; Alola et al. 2020).

In the US, the Federal Reserve executes five main functions to maintain economic stability: conducting monetary policy, promoting financial system stability, supervising and regulating financial institutions and activities, fostering payment and settlement system safety and efficiency, and promoting consumer protection and community development (Federal Reserve 2022). As exchange rates are key determinants of tourism demand, the tourism industry cannot work in isolation from the country’s monetary policy stance (See Usman et al. 2022). Hence, exchange rate and monetary policy uncertainty dynamics between the US and tourism destinations drive tourism development vis-à-vis the restaurant and hotel industry’s expansion.

Previous studies have examined the impact or nexus between exchange rate and tourism activities (Tang 2013; Alola et al. 2019; Usman et al. 2022). Tang (2013) found a short- and long-run Granger causality from exchange rates in real tourism receipts. Falk (2015) found that, especially during the winter season, Swiss overnight visitors in Western Austrian Ski resorts respond sensitively to exchange rate dynamics. Monetary policy dynamics have also consistently been linked to tourism activities (Chen 2010). Usman et al. (2021) examined the exchange rate pass-through to restaurant and hotel prices using a linear model that assumes that prices of restaurants and hotels react identically to positive and negative exchange rate fluctuations. Monetary policy uncertainty is increasing in the US, which may affect the prices of hospitality-related services. Moreover, positive exchange rate or monetary policy uncertainty shocks may behave differently from negative shocks of identical size.

In this study, we investigate whether an asymmetric pass-through of the exchange rate to restaurant and hotel prices exists while accounting for monetary policy uncertainty in the US. Hence, we incorporate the global price of energy (EPR) as a control variable in the hotel price model.^1^ In 2021, US fast food chains emerged as the first among top eight performing industries locally, and twentieth among the total of twenty-five global performing restaurants (Brandirectory 2021). Moreover, Brandirectory (2021) noted that despite COVID-19 pandemic-related disruptions, major food chains exhibited remarkable adaptability, minimizing the pandemic-induced shocks and damage to the subsector. Hence, this study presents a significant extension of literature on tourism development for obvious reasons. First, considering that the US is a world-leading market for global fast food, restaurant, and hotel brands, this study specifically focuses on the restaurant and hotel industries and consists a considerably rare study on the macro-level of business and the economy. Second, this study employs a nonlinear autoregressive distributed lag (NARDL) modeling technique with a pass-through perspective that completely illustrates both the dimensions and directions of exchange rate shocks on restaurant and hotel service prices.

The succeeding sections are organized as follows. Section 2 presents a literature review. Section 3 describes the adopted dataset and empirical methods. Sections 4 and 5 discuss the results and conclusions of the study.

## Literature review and research hypothesis development

### Theoretical literature

Exchange rate pass-through is theoretically embedded in purchasing power parity, derived from the law of one price. Purchasing power parity theory was first proposed in the sixteenth century at the University of Salamanca, while its modern version was popularized in 1916 by Swedish economist Gustav Cassel. This theory states that at equilibrium level, the market prices of tradable goods and services remain identical in different countries if goods and services prices are measured according to an identical unit of currency. Purchasing power parity theory is based on and follows perfect and existing competitive arbitrage activities, which compel exchange rates to adjust toward equilibrium, given no transport costs, tariffs, and imperfect competition. However, empirical studies have demonstrated that purchasing power parity or law of one price, either in its absolute or relative versions, does not hold owing to the stickiness of nominal prices resulting from weak competitive arbitrage activities (Balcilar et al. 2020).

By eliminating economic arbitrage activities, we have developed several theoretical models to address the difficulty and cost of achieving a unanimous agreement. Hence, researchers have emphasized a shift toward soft consensus models (see Kuo et al. 2014, 2016; Zhang et al. 2019). By proposing soft consensus cost models for group decision-making based on loan consensus in online P2P lending, Zhang et al. (2019) demonstrate that P2P lending is beneficial to both borrowers and lenders by eliminating middlemen and their arbitrage activities, which reduces risks and maximizes returns. Moreover, Chao et al. (2021) apply a large-scale group decision-making model with cooperative behaviors and heterogeneous preferences in financial inclusion. Their experimental results indicate that by comparing a model’s performance with that of an existing model through a poverty reduction-targeted project in China, the efficacy of the proposed model can be validated owing to the difficulty in selecting beneficiaries in financial inclusion. This is because they lack not only credit history but a large number of participants, and participants have mixed views.

### Empirical literature

Destination or border prices of commodities—especially tourism-related services—are competitively driven by both domestic and international factors (Dwyer et al. 2002). Campa and Goldberg (2005) and Usman et al. (2021) explored this observation further in the tourism industry by evaluating the existence of a pass-through effect of the exchange rate on tourism-related prices. In this section, we review existing studies with relevant hypotheses.

#### Exchange rates and restaurant-hotel prices

A study on the nexus of exchange rate and restaurant price by Fullerton et al. (2009) analyzed restaurant prices of eight international border businesses or franchises in El Paso, Texas, Ciudad, and Juarez. Employing seven and one US and Mexican multinational corporations or affiliate, respectively, Fullerton et al. (2009) surveyed the prices of 32 menu items, yielding a total of 132 for each pair of prices as the number of sampled observations. This study demonstrated that the price ratio of menu items in international restaurants in Ciudad, Juarez, El Paso, and Texas exhibit strong correlations with the peso/dollar exchange rate. Moreover, an exploratory analysis revealed a significant but very short half-life deviation for eight different products. Similarly, Tang (2015) employed a dataset of publicly traded restaurant firms over the period 1990–2012 in the US, which covered three business cycles. The study examines (i) the determinants of risk exposure and (ii) degree of risk exposure to commodity prices in the restaurant industry. While utilizing the modeling of the determinants of equity risk exposure via the discounted cash flow approach, 60-month rolling regression accounts for the risk exposure of the equity returns were estimated. Notably Tang (2015) showed that commodity price risk was confirmed in 35.39% of the sampled restaurant businesses. In these business, levels of equity risk exposure associated with periods of price booms and slumps were 1.148 and 1.031, respectively. However, more study findings revealed that while operating and financial leverages could minimize risk exposure, these could be ineffective tool during commodity price booms and slumps owing to asymmetric effects.

Moreover, Aalen et al. (2019), building on existing literature gaps, examined the extent to which exchange rate affects inbound hotel demand. Using Norway as a destination country, ten different source countries—Denmark, France, Germany, Italy, Japan, the Netherlands, Spain, Sweden, the United Kingdom, and the US—were examined over a 2007–2015 period. Using a panel of monthly hotel accommodations sold in the destination country to potential visitors from the aforementioned source countries, the study revealed that inbound hotel demand responded with an equal amount to the bed prices (i.e., a unitary elastic). Balcilar et al. (2020), using Nigerian time series data on quarterly frequency, found that exchange rate to prices pass-through is incomplete with evidence that the long-run pass-throughs are stronger than short-run pass-throughs. In a related US case, Usman et al. (2021) reported that an exchange rate appreciation affects restaurant and hotel prices but increased prices in energy and tourism development are responsible for restaurant and hotel price shocks in the US based on quarterly time series data over a 2001(Q4)–2017(Q4) period.

Thus, we present the following hypothesis:

##### H_1_

Pass-through of the exchange rate to restaurant and hotel prices is asymmetric in the US.

#### Monetary policy and restaurant-hotel prices

Extant studies have revealed that monetary policy administered by apex banks exerts a varying degree of effects on all economic sectors, including hospitality-related sectors (Chen 2010, 2012; Chen et al. 2010). However, most studies addressed the effect of monetary policy on the hospitality industry with a holistic approach (i.e., without considering the specificity of the restaurant and hotel prices). Chen (2010) and Chen et al. (2010) examined a shifting effect from the monetary policy of different economies. While Chen et al. (2010) examined the monetary effects associated with the stock performance in the airlines, hotels, restaurants, and tourism-related businesses, Chen (2010) outlined the same objective for the US. Chen (2010) classified changes in the discount or federal fund rates as either expansionary (for an expansive period) and contractionary (for a restrictive period) monetary policy tools respectively. While this study revealed that the monetary policy dimensions exert varying degrees of impact, the authors observed important changes due to the federal fund rates in the stock returns of the country’s restaurants with the discount rates causing any significant change in the hospitality stock prices. Chen et al. (2010) confirmed that discount rate a decrease (expansive monetary policy) significantly affected hotel and tourism stocks in Hong Kong.

Moreover, studies by Chen et al. (2012) and Fougère et al. (2010) have presented another dimension with determinants of restaurant and hospitality-related prices. Fougère et al. (2010) examined the observation in the Japanese hotel stock returns by exploring series of macroeconomic variables including percentage changes in money supply, unemployment, consumer price index (CPI), industrial production, oil price, total trade, and yen-dollar exchange rate alongside discount rate changes. Notably, the study outlined that changes in discount rate, unemployment rate, and oil prices can cause significant impact on national hotel stock returns, thus posing as determinants of the industry stock market. Similarly, while examining key determinant(s) of restaurant prices, Chen et al. (2012) estimated CPI from the individual price quotes and examined how minimum wages affect restaurant prices in France. Despite establishing a positive relationship between restaurant prices and minimum wages in the country, this study revealed that changes in minimum wage mostly pass through retail prices in not less than 1 year.

##### H_2_

Pass-through of monetary policy to restaurant and hotel prices is asymmetric in the US

Munir and Iftikhar (2021), Irandoust (2019), and Ongan et al. (2018) have examined an empirical connection between exchange rates and tourism and recreation activities. For instance, Munir and Iftikhar (2021), while employing a hidden cointegration analysis within a likelihood-based panel framework for 10 European countries, examined the asymmetric effect of exchange rate on tourism demand. The investigation affirmed that tourism demand responds asymmetrically to the exchange rate fluctuations especially in the long run, further suggesting that depreciation and appreciation of exchange rates affect tourism demand in different dimensions.

##### H_3_

Pass-through of energy prices to restaurant and hotel prices is asymmetric in the US.

#### Energy prices and restaurant-hotel prices

Studies on the effect of energy prices on tourism development have used oil prices as a proxy for energy prices (See Balcilar et al. 2022). Using the Bayesian vector autoregression with stochastic volatility, Clark and Terry (2010) showed that core inflation responded significantly to energy price shocks at the beginning of 1975 in the US. This response declined sharply and remained low. However, with effective monetary policy, responsiveness to energy inflation has decreased since 1985. Similarly, using the NARDL model, Lacheheb and Sirag (2019) examine the pass-through of oil price shocks to inflation in Algeria. Their empirical results suggested evidence of a nonlinear effect of oil price on inflation and further demonstrated that oil price does not have a significant relationship with inflation in Algeria. Moreover, using the NARDL model and an asymmetric causality test, Usman et al. (2020) detected an asymmetric pass-through of energy prices to US inflation. Moreover, they noted an asymmetric causal relationship flowing from positive and negative shock in energy prices to positive and negative shock in inflation. Sek (2022) assessed how oil price changes affect sectoral inflation in Malaysia. Results based on the Markov-Switching model suggest an asymmetric oil price effect on price inflation, industrial production, and producer price. The study showed that the effects of oil prices on industrial production and producer prices are quite stronger than that of other investigated indicators. Moreover, sectors linked to energy resources tend to experience a higher effect of oil prices on CPI, industrial production, and producer prices. By recognizing a significant amount of carbon emission from the transportation industry to the atmosphere, Kou et al. (2022) extend group decision-making and spherical fuzzy numbers to provide strategies to stimulate the effectiveness of solar energy investment projects. This novel methodology is based on hybrid decision-making, and the results suggested that dynamicity is the most critical TRIZ-based factor, and composite materials, with a weight of 0.255, have a critical impact. The study concluded solar panels have to be designed vertically to receive sunlight at different periods.

According to the previous studies, including the work of Usman et al. (2021) which is closer in perspective, no studies considered the role of monetary policy while examining the asymmetric role of the exchange rate in tourism-related aspects’ development. Particularly, Usman et al. (2021) failed to account for possible asymmetries in the exchange rate-tourism price nexus for the US.

## Data source and methodology

### Data and source

In this study, we employed the logarithmic transformation of the US monetary policy uncertainty (MP), nominal effective exchange rate (NEER), restaurant and hotel prices (RHP) measured as the harmonized index of consumer prices for the US, index (2015 = 100), and the global price of energy index, (Index 2016 = 100) for the period 2001:M12 to 2019:M01. Notably, we retrieve NEER data from International Financial Statistics database of the IMF. We retrieved RHP and the global price of energy index from the Federal Reserve Economic Data of the Federal Reserve Bank of St. Louis, while MP is obtained from the Economic Policy Uncertainty Database.

### Methodology

Price can react directly to exchange rate shocks, which is a central focus of the purchasing power parity (PPP) doctrine (Balcilar et al. 2021a, b; Usman 2020). In this study, we extend the original PPP equation, which shows the nexus between exchange rate and prices, by augmenting shocks to monetary policy uncertainty using the NARDL approach. Conversely, positive and negative partial sums of the explanatory variables are derived from the following decomposition:

1 $$X_{t}^{ + } = \mathop \sum \limits_{j = 1}^{t} \Delta X_{j}^{ + } = \mathop \sum \limits_{j = 1}^{t} \max \left( {\Delta X_{j} ,0} \right)\;{\text{and}}\;X_{t}^{ - } = \mathop \sum \limits_{j = 1}^{t} \Delta X_{j}^{ - } = \mathop \sum \limits_{j = 1}^{t} \min \left( {\Delta X_{j} ,0} \right)$$

Here, explanatory variables $X_{j}^{ + }$ and $X_{j}^{ - }$ in Eq. (1) represent the positive and negative exchange rate fluctuations (NEER), monetary policy uncertainty (MP), and global price of energy (EPR). Following Shin et al. (2014), we specify the NARDL model as follows:

2 $$\begin{aligned} \Delta lnRHP_{t} = & \gamma + \theta_{0} lnRHP_{t - 1} + \beta_{1}^{ + } lnNEER_{t - 1} + \beta_{1}^{ - } lnNEER_{t - 1} + \beta_{2}^{ + } lnMP_{t - 1} \\ & \quad + \beta_{2}^{ - } lnMP_{t - 1} + \beta_{3}^{ + } lnEPR_{t - 1} + \beta_{3}^{ - } lnEPR_{t - 1} + \mathop \sum \limits_{j = 1}^{p} \lambda_{0,j} \Delta lnRHP_{t - j} \\ & \quad + \mathop \sum \limits_{j = 0}^{q} \varphi_{1,j} \Delta lnNEER_{t - j}^{ + } + \mathop \sum \limits_{j = 0}^{q} \varphi_{1,j} \Delta lnNEER_{t - j}^{ - } + \mathop \sum \limits_{j = 0}^{q} \varphi_{2,j} \Delta lnMP_{t - j}^{ + } \\ & \quad + \mathop \sum \limits_{j = 0}^{q} \varphi_{2,j} \Delta lnMP_{t - j }^{ - } + \mathop \sum \limits_{j = 0}^{q} \varphi_{3,j} \Delta lnEPR_{t - j}^{ + } + \mathop \sum \limits_{j = 0}^{q} \varphi_{3,j} \Delta lnEPR_{t - j}^{ - } + \varepsilon_{t} \\ \end{aligned}$$

where ln denotes the logarithmic transformation of the variables $\gamma$ and represents the model intercept, while $\beta$ and $\varphi$ represent slopes of the long-run and short-run coefficients. Terms p and q denote orders of lags used for the estimation. Following the empirical studies of Delatte and Lopez-Villavicencio (2012), we include the global price of energy as a control variable to determine restaurant and hotel price changes in the US. Furthermore, $\varepsilon_{t}$ is the error term, which follows a stochastic Gaussian process with zero-mean and variance $\sigma^{2}$, $\varepsilon_{it} \sim iid\left( {0,{\upsigma }^{2} } \right)$. Hence, procedures for the estimations are summarized as (**I**) testing the stationarity properties of the series for the avoidance of I(2) in the series, (**II**) testing the short-run asymmetry $(\varphi_{i}^{ + } = \varphi_{i}^{ - } )$ and long-run asymmetry $(\beta_{i}^{ + } = \beta_{i}^{ - } )$ by employing the standard Wald test, $i = 1,2,3$,^2^ (**III**) testing the null hypothesis of no cointegration $\beta_{i} = { }\beta_{i}^{ + } = \beta_{i}^{ - } = 0$ using *F*-statistic and *t*-statistic, and (**IV**) the long-run asymmetric coefficient is estimated as $L\psi_{i}^{ + } - \frac{{\beta_{i}^{ + } }}{{\theta_{0} }}$ and $L\psi_{i}^{ - } - \frac{{\beta_{i}^{ - } }}{{\theta_{0} }}$, where $L\psi_{i}^{ + }$ and $L\psi_{i}^{ - }$ denote the positive and negative long-run coefficients, while the positive and negative short-run coefficients are represented by $\varphi_{i}^{ + } {\text{and }} \varphi_{i}^{ - }$, respectively.

Additionally, to examine the asymmetric causality between the variables, we perform an asymmetric causality test developed by Hatemi (2012). The asymmetric causality employed in this study considers the positive and negative shocks between two integrated variables. Specifically, the cumulative form in Eq. 1 is used to investigate the asymmetric causal relationship between the variables through a vector autoregressive model of order *p*, vector autoregression VAR (*p*) as suggested by Hatemi (2012).

## Results and discussion

**Fig. 1:**
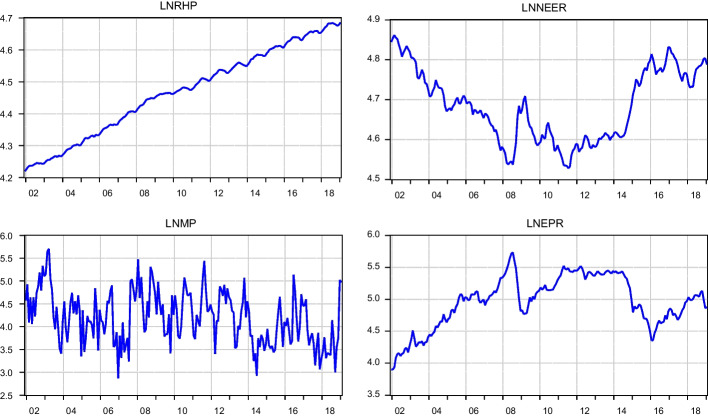
Time series plot of variables for the study

We first assessed the visual properties of the series employed. Essentially, we examined time plots of the series against the possibility of drift, seasonality, trend, and structural breaks. Figure 1 indicates that the log of the RHP slopes upward, which suggests that variables increased over the years covered. We characterized NEER and monetary policy uncertainty by fluctuations with no evidence of a particular trending pattern. Conversely, the log of energy price, although associated with structural breaks, trends upward after a global financial crisis. Breaks found in the series can be partly attributed to macroeconomic policy changes. Exchange rate and energy price graphs spikes in 2008 may be attributed to the global financial crisis that started in the US toward the end of 2007. This crisis disrupted the US dollar and consequently affected global energy prices. Moreover, crude oil prices fell considerably between 2014 and 2016, which subsequently decreased energy prices.^3^ Furthermore, fluctuations in variables are more conspicuous in the case of monetary policy uncertainty for two main reasons: first, the variable is already an uncertainty variable; second, monetary policy rate is frequently adjusted to solve the country’s macroeconomic problems.

Table 1 depicts the descriptive statistics of the variables explored in this study. The average values of the variables in their natural logarithms are 4.468 for lnRHP, 4.684 for lnNEER, 4.1999 for lnMP, and 4.963 for energy price. Values of the standard deviation are less than 1 in all the variables, which suggests that the variables exhibit a low volatility level. The values of skewness of the variables are not far from zero in all variables. Hence, frequency distribution is considerably close to symmetry. lnRHP and lnEPR present a negative skewness, while lnNEER and lnMP present positive skewness. Furthermore, the kurtosis values for all variables indicate a flat-topped (platykurtic), and values for the Jarque–Bera statistics are high for all variables except lnMP. Hence, the null hypotheses of the normal distribution is rejected for all variables except lnMP. This implies that the distribution of the variables explored is not normal except for lnMP, which depicts a normal distribution.

**Table 1:** Descriptive statistics

	LNRHP	LNNEER	LNMP	LNEPR
Mean	4.468430	4.684192	4.199902	4.963228
Median	4.474834	4.679676	4.195268	4.999689
Maximum	4.685459	4.861645	5.706278	5.734042
Minimum	4.220096	4.528277	2.868820	3.899097
SD	0.136999	0.089364	0.578479	0.405442
Skewness	− 0.164492	0.123874	0.119939	− 0.485736
Kurtosis	1.838216	1.804422	2.393804	2.549732
Jarque–Bera	12.51427	12.79591	3.648041	9.840797
Probability	0.001917	0.001665	0.161376	0.007296
Sum	920.4965	964.9435	865.1798	1022.425
Sum Sq. Dev	3.847607	1.637131	68.60066	33.69851
Observations	206	206	206	206

Authors’ computation

Next, we test whether a nonlinear model is appropriate for this study. Hence, we conduct two different symmetric tests. The first test considers long- and short-run asymmetry differently using the standard Wald test. Panel A of Table 2 indicates that the results provide evidence that the null hypothesis of symmetric relationship is rejected in all cases, except the short run for lnMP. In the second test, we use the Broock, Scheinkman, and Dechert (BDS) linearity tests proposed by Brock et al. (1996). This test uses the residuals of dynamic interactions among the variables. Results in Table 2, Panel B, demonstrate that the null hypothesis, wherein the residuals of the model are independently and identically distributed $\left(i.i.d\right),$ is rejected. This implies that the relationship between the variables is characterized by nonlinearity. From these findings, we conclude that the dynamic relationship estimated in this study includes nonlinear characteristics. Therefore, nonlinear model can better produce robust findings for policy formulations.

**Table 2:** Symmetry tests

Exogenous	Long-run Asymmetry (W_LR_)		Short-run Asymmetry (W_SR_)	
Variable	F-Statistic	*P*-value	F-Statistic	*P*-value
Panel A: Long-run and short-run symmetric tests				
lnNEER_*t*_	9.652***	0.000	7.7778***	0.000
lnMP_*t*_	91.15***	0.000	1.138	0.288
lnEPR_*t*_	6.049***	0.000	4.413**	0.037

Variable	BDS Statistic	SE	*P*-value
Panel B: BDS non-linearity tests			
lnRHP_*t*_	0.2040***	0.0029	0.0000
lnNEER_*t*_	0.1836***	0.0029	0.0000
lnMP_*t*_	0.0515***	0.0035	0.0000
lnEPR_*t*_	0.1830***	0.0040	0.0000

** and *** denote 1% and 5% significance levels. W_LR_ and W_SR_ indicate the Wald test for the long- and short-run with their respective *P*-values

Superscript ***shows a significance level at 0.01 with a maximum cor. dimension of 2

Furthermore, we test for the unit root in the series explored by first applying the standard unit root tests via the augmented Dickey-Fuller (ADF), Kwiatkowski-Phillips-Schmidt-Shin (KPSS), and the Phillips-Perron (PP) tests. Table 3 indicates that both RHP and exchange rates are not stationary at levels except after their first differences. Monetary policy and energy prices remain stationary both at their levels and first differences. To circumvent the effect of structural breaks that may affect test outcomes, we apply a structural break unit root test from Lee and Strazicich (2013). Hence, results in Table 4 indicate that except for monetary policy uncertainty, which is stationary at levels, variables including RHP, exchange rate, and energy prices remain stationary after their first differences. This means that the series has a unit root with a break that cannot be held for these variables except monetary policy uncertainty, which is stationary at levels. These results imply that in this study, there is a mixed order of integration in the variables explored (i.e., I(0) and I(1)). This means that we can proceed with the estimation of our NARDL model.

**Table 3:** Unit root tests

Variable	ADF test		KPSS test		PP test	
	Model A	Model B	Model A	Model B	Model A	Model B
Panel A: Ln Level Series						
lnRHP_*t*_	− 1.2343	− 2.6658	1.8060**	0.3740***	− 1.1361	− 2.6178
lnNEER_*t*_	− 2.1265	− 2.1205	0.4080*	0.4081***	− 1.8183	− 1.7359
lnMP_*t*_	− 6.7715***	− 7.1822***	0.5875**	0.1000***	− 6.7036***	− 7.1358***
lnEPR_*t*_	− 2.7919*	− 2.3928	0.5719**	0.3530***	− 2.6847*	− 2.2373
Panel B: Ln First Difference Series						
∆lnRHP_*t*_	− 2.2047	− 2.4143	0.1116	0.0209	− 9.0534***	− 9.0257***
∆lnNEER_*t*_	− 9.2629***	− 9.2828***	0.3594*	0.0378	− 9.3790***	− 9.5511***
∆lnMP_*t*_	− 19.227***	− 19.195***	0.1726	0.1178***	− 29.084***	− 28.189***
∆lnEPR_*t*_	− 10.166***	− 10.305***	0.2857	0.0434	− 10.179***	− 10.321***

This table reports the results of the augmented Dickey-Fuller (ADF), Kwiatkowski-Phillips-Schmidt-Shin (KPSS), and Phillips-Perron (PP) unit root tests. Model A includes only a constant as a deterministic component in tests regression; model B includes both a constant and a linear time trend. The null hypothesis for the ADF and PP tests simply states that the series is nonstationary, but it is stationary in the case of the KPSS test. ***, **, and *denote significance at 1%, 5%, and 10% levels respectively. Ln denotes the natural logarithm of the series. We selected an automatic lag length of max. (10) based on Schwarz Information Criterion

**Table 4:** Lee-Strazicich Unit Root Test with Structural Breaks

Variables	L-S test at level		L-S test at 1st difference	
	Statistics	Break date	Statistics	Break date
lnRHP_*t*_	− 1.2469 (8)	2008:M08	− 10.1733 (8)***	2008:M12
lnNEER_*t*_	− 1.3172 (7)	2011:M09	− 4.5545 (6)***	2003:M06
lnMP_*t*_	− 5.3224 (1)***	2014:M03	− 5.6637 (3)***	2009:M02
lnEPR_*t*_	− 1.9019 (2)	2004:M09	− 9.9523 (0)**	2004:M03
Critical values				
1 Percent	− 4.0497		− 4.0491	
5 Percent	− 3.4517		− 3.4510	
10 Percent	− 3.1465		− 3.1458	

^**^ and ***denote 1% and 5% significance levels. The lag length is given in the bracket (). The null hypothesis is that the series has a unit root with a break

Prior to model estimation, we conduct a series of diagnostic tests (Table 5). Estimated model residuals show that the Breusch-Godfrey Lagrange multiplier test for serial correlation, Breusch-Pagan-Godfrey conditional heteroskedasticity test, Ramsey regression equation specification error test (RESET) test, and Jarque–Bera normality test. As autocorrelation, heteroscedasticity, functional misspecification were not found, our results suggest that the NARDL model for this study is correctly specified. Moreover, the model residuals are normally distributed.

**Table 5:** Diagnostic tests

Model diagnostics	F-Statistic	*P*-value
*χ*_SC_^2^	1.6397: [1]	0.5286
*χ*_H_^2^	1.0918: [1]	0.2973
*Χ*_FF_^2^	0.4552: [1]	0.6495
*χ*_N_^2^	1.7100	0.4253

*χ*_SC_^2^ denotes the Breusch-Godfrey Serial Correlation LM test, *χ*_H_^2^ denotes the Breusch-Pagan-Godfrey conditional heteroskedasticity test, *χ*_FF_^2^ denotes the Ramsey RESET test, and *χ*_N_^2^ denotes the Jarque–Bera normality test. The values in [] represent the number of lags selected

Table 6 presents the results of the long- and short-run asymmetric effects of exchange rate and monetary policy uncertainty on RHP. Before discussing the long-run and short-run coefficients, we present the results of the asymmetric cointegration tests, which are based on the bounds-testing approach. This test is a modified version of the F-statistic proposed by Pesaran et al. (2001) and the t-statistic by Banerjee et al. (1998). Test results indicate the values of the F-stat and t-stat (i.e., 6.4528 and − 5.7596) are greater than the critical values at a 1% significance level (Table 6). In the presence of structural breaks, cointegration exists between dependent and explanatory variables.

**Table 6:** NARDL long- and short-run coefficients

Variable	Long-run coefficients		Short-run coefficients	
	Coefficient	SE	Coefficient	SE
Model Selection: (2, 3, 3, 0, 1, 0, 2)				
lnRHP_*t*−1_			1.2851***	0.0638
lnRHP_*t*−2_			− 0.4813***	0.0659
lnNEER_*t*_^+^	− 0.0868***	0.0231	− 0.0913**	0.0401
lnNEER_*t*−1_^+^			− 0.1335**	0.0505
lnNEER_*t*−2_^+^			0.0697**	0.0321
lnNEER_*t*−3_^+^			− 0.0446	0.0345
lnNEER_*t*_^−^	0.1697***	0.0307	0.1334**	0.0513
lnNEER_*t*−1_^−^			0.0693	0.0494
lnNEER_*t*−2_^−^			− 0.1175**	0.0491
lnNEER_*t*−3_^−^			0.0807**	0.0337
lnMP_*t*_^+^	− 0.0729**	0.0284	− 0.0358***	0.0052
lnMP_*t*_^−^	0.0807**	0.0314	0.0392***	0.0086
lnMP_*t*−1_^+^			0.00176*	0.00089
lnEPR_*t*_^+^	0.0127**	0.0058	0.0197***	0.0024
lnEPR_*t*_^−^	− 0.0090	0.0057	− 0.0007	0.0012
lnEPR_*t*−1_^−^			− 0.0039	0.0094
lnEPR_*t*−2_^−^			0.0027	0.0062
ECM_*t*−1_			− 0.1962***	0.0304
Constant			0.8274***	0.1405
*F*_PSS_	6.4528***			
*t*_BDM_	− 5.7596***			

Superscripts *, **, and *** represent level of significance at 1%, 5%, and 10%. *F*_PSS_ uses F-statistic and *t*_BDM_ uses a t-statistic

Notably, in the long run, a 1% positive shock to the exchange rate reduces RHP by 0.0868%. Conversely, a negative shock of the same magnitude increases RHP by 0.1697%. Both coefficients of positive and negative shocks in the exchange rate, in the long run, are statistically significant. Plausible economic reasons for these results are based on the fact that domestic currency appreciation inhibits exports and reduces imports price. Conversely, domestic currency depreciation stimulates exports and increases imports price. Hence, as domestic currency appreciates, prices of restaurants and hotels tend to decrease as imported commodities, including inbound tourism, become more expensive. Furthermore, a close examination of these results reveals that the pass-through of a depreciation of the exchange rate is stronger in magnitude than the pass-through of an appreciation of the exchange rate. Furthermore, in the short run, the pass-through of a 1% positive shock in the exchange rate to RHP is negative (i.e., − 0.0913%). Conversely, a 1% negative shock in the exchange rate increases RHP by 0.1334%. Pass-through is stronger in for depreciating exchange rates than in appreciating exchange rates. This finding is consistent with Usman (2020), who found evidence of a stronger pass-through effect for exchange rate depreciation. Moreover, our finding is consistent with Balcilar et al. (2020), who found evidence that exchange pass-through is larger in magnitude in the long run than the pass-through in the short run for Nigeria.

Our results indicate that a positive shock to monetary policy uncertainty causes RHP to fall significantly in the long run and short run. Conversely, a similar negative shock would cause RHP to increase with evidence of statistical significance only in the long run. Specifically, the magnitude of a 1% positive shock to monetary policy uncertainty significantly reduces RHP in the long run by roughly 0.0729%. Conversely, that of a 1% negative shock to monetary policy uncertainty significantly increases RHP by about 0.0807%. In the short run, our results indicate that positive shocks to monetary policy uncertainty would significantly result in a decline in RHP in the US by 0.0358%. However, a 1% negative shock in monetary policy uncertainty increases RHP significantly by 0.0392%. Our findings indicate that a high level of uncertainty reduces inbound tourism, which consequently results in a fall in RHP. However, when level of uncertainty shocks reduces, inbound tourism would increase. This increase may trigger restaurant and hotel owners to increase prices. This evidence is similar to Chen (2010), who found that an expansive monetary policy significantly impacted the hotel and tourism stocks in Hong Kong.

Furthermore, we incorporate energy prices as determinants of RHP. Our results show that in the long run (short run), a 1% positive shock in energy prices increases RHP by 0.0127% (0.0197%). Conversely, in both the long and short run, a 1% negative shock in energy prices is negative and insignificantly related to the prices of restaurant and hotel. This suggests that US energy prices are inflationary in the restaurant and hotel industry only in the long run. Hence, as energy prices increase, restaurants and hotel prices increase owing to the industry’s dependence on large amounts of energy in its operations. Moreover, the insignificant effect of a negative shock in energy prices suggests that RHP only respond to increases in energy prices. Furthermore, the coefficient of the error correction term (ECT) (− 0.1962) implies that RHP converge to the long-run equilibrium level by a 19.6% adjustment speed every month through positive and negative shocks in the exchange rate, monetary policy uncertainty, and energy prices.

To craft appropriate macroeconomic policies to sustain low and stable price levels in the US restaurant and hotel industry, we employ a nonlinear causality test proposed by Hatemi-J (2012). This test considers the asymmetric causal relation between two variables within the framework of Toda and Yamamoto’s (1995) causality. We use the Hatemi-J Criterion (HJC) for lag selection. Our results indicate that the null hypothesis of a positive shock in exchange rate not causing a positive shock in RHP is rejected at a 10% level of significance (Table 7). However, negative shocks in the exchange rate causing negative shock in RHP is unsupported, consistent with Aalen et al. (2019) who find equal responses of exchange rate to hotel prices in 10 countries. Our results do not detect any causality from lnRHP and exchange rate and vice versa.

**Table 7:** Asymmetric causality test

Null hypothesis	*P*-value	Null hypothesis	*P*-value
lnNEER^+^ ≠ > lnRHP^+^	0.086	lnRHP^+^ ≠ > lnNEER^+^	0.743
LnNEER^−^ ≠ > lnRHP^−^	0.769	LnRHP^−^ ≠ > lnNEER^−^	0.516
LnMP^+^ ≠ > lnRHP^+^	0.536	LnRHP ≠ > lnMP	0.201
LnMP^−^ ≠ > lnRHP^−^	0.987	LnRHP^−^ ≠ > lnMP^−^	0.878
LnEPR^+^ ≠ > lnRHP^+^	0.027	LnRHP^+^ ≠ > lnEPR^+^	0.920
LnEPR^−^ ≠ > lnRHP^−^	0.538	LnRHP^−^ ≠ > lnEPR^−^	0.778
LnMP^+^ ≠ > lnNEER^+^	0.002	LnNEER^+^ ≠ > lnMP^+^	0.072
LnMP^−^ ≠ > lnNEER^−^	0.092	LnNEER^−^ ≠ > lnMP^−^	0.088
LnEPR^+^ ≠ > lnNEER^+^	0.953	LnNEER^+^ ≠ > lnEPR^+^	0.092
LnEPR^−^ ≠ > lnNEER^−^	0.349	LnNEER^−^ ≠ > lnEPR^−^	0.035

≠  > denotes the null hypothesis of no causality. Hatemi-J Criterion (HJC) is used for lag selection

Furthermore, our results find that a positive or negative shock in monetary policy uncertainty does not cause positive or negative RHP in a Granger sense. Similarly, a positive or negative shock in RHP does not cause a positive or negative shock in monetary policy uncertainty. While a positive energy price shock causes a positive restaurant and hotel price shock, there is no evidence to support that a negative shock in energy price Granger-causes a negative shock in RHP. Moreover, we could not detect any evidence to support that either positive or negative shocks in RHP cause energy price shocks.

Results of the asymmetric causality between monetary policy uncertainty and exchange rate present a feedback effect. The null hypothesis that a positive (negative) shock in monetary policy uncertainty not causing a positive (negative) exchange rate can be rejected at a 1% and 10% significance level. Similarly, the null hypothesis that a positive (negative) shock in exchange rate does not cause monetary policy uncertainty can also be rejected at a 10% significance level. These results imply that both monetary policy uncertainty and exchange rate shocks can be used to predict each other. Regarding asymmetric Granger causality between energy price and exchange rate, the null hypothesis that a positive (negative) shock in energy price does not cause exchange rate cannot be rejected. However, the null hypothesis that a positive (negative) shock in exchange rate not causing energy prices is rejected. Therefore, a one-way asymmetric causality moves from a positive shock in the exchange rate to that in energy price and from a negative shock in the exchange rate to that in energy price.

Given the discussion of the estimated results, it is evident that pass-through of exchange rate and monetary policy uncertainty to RHP is asymmetric, and the coefficient of both negative and positive shocks is inelastic and statistically significant. Hence, hypotheses (1) and (2) are supported by the empirical results of this study. Furthermore, for hypothesis (3), empirical results provide the support that the pass-through of energy price to restaurant and hotel price is asymmetric, but the coefficient of positive change in energy price is only statistically significant. This implies that the third hypothesis is not supported by empirical evidence.

### Robustness check

To determine the robustness of our estimations, we capture the effect of structural breaks identified in the series via the NARDL modeling technique. Table 8 results suggest that in the presence of structural breaks, the Breusch-Godfrey LM test for serial correlation, Breusch-Pagan-Godfrey conditional heteroskedasticity test, Ramsey RESET test, and Jarque–Bera normality test provide the best model fit. Moreover, Table 9 indicates that all coefficients survive. Effects of structural breaks are not statistically significant in the model. Additionally, the ECT coefficient is − 0.2878, which suggests that RHP in the US converge to their long-run equilibrium level by a 28.8% adjustment speed every month. This is possible through positive and negative shocks in the exchange rate, monetary policy uncertainty, and energy prices.

**Table 8:** Diagnostic tests

Model diagnostics	F-Statistic	*P*-value
*χ*_SC_^2^	1.3611: [2]	0.2589
*χ*_H_^2^	1.4126: [1]	0.5214
*Χ*_FF_^2^	1.0095: [1]	0.3141
*χ*_N_^2^	3.4672	0.1766

*χ*_SC_^2^ denotes the Breusch-Godfrey Serial Correlation LM test, *χ*_H_^2^ denotes the Breusch-Pagan-Godfrey conditional heteroskedasticity test, *Χ*_FF_^2^ denotes the Ramsey RESET test, and *χ*_N_^2^ denotes the Jarque–Bera normality test. Values in [] represent the number of lags selected

**Table 9:** NARDL long- and short-run coefficients

Variable	Long-run coefficients		Short-run coefficients	
	Coefficient	SE	Coefficient	SE
Model selection: (2, 2, 0, 0, 3, 0, 3)				
lnRHP_*t*−1_			1.1971***	0.0668
lnRHP_*t*−2_			− 0.4428***	0.0664
lnNEER_*t*_^+^	− 0.2732***	0.0581	− 0.0108***	0.0031
lnNEER_*t*−1_^+^			− 0.0627*	0.0322
lnNEER_*t*−2_^+^			− 0.0692	0.0551
lnNEER_*t*_^−^	0.4899***	0.0615	0.0347**	0.0141
lnMP_*t*_^+^	− 0.0262***	0.0089	− 0.0210***	0.0053
lnMP_*t*_^−^	0.1592***	0.0499	− 0.0455***	0.0082
lnMP_*t*−1_^−^			0.0018*	0.0010
lnMP_*t*−2_^−^			− 0.0017*	0.0009
lnMP_*t*−3_^−^			0.0015	0.0095
lnEPR_*t*_^+^	0.4813***	0.0627	0.0232**	0.0096
lnEPR_*t*_^−^	− 0.1281	0.0809	− 0.0154	0.0565
lnEPR_*t*−1_^−^			− 0.1098	0.0853
lnEPR_*t*−2_^−^			0.1647*	0.0866
lnEPR_*t*−3_^−^			− 0.1654	0.1066
*D*_2004:09			− 0.00398	0.0033
*D*_2008:08			− 0.00597	0.0043
*D*_2011:09			0.00011	0.0032
*D*_2014:03			0.00443	0.0031
ECM_*t*−1_			− 0.2878***	0.0391
Constant			1.0378***	0.1528
*F*_PSS_	6.5388***			
*t*_BDM_	− 4.7352***			

Superscripts *, **, and *** represent level of significance at 1%, 5%, and 10%. *F*_PSS_ uses F-statistic and *t*_BDM_ uses t-statistic

## Conclusion and policy implications

The US serves as a major host and world-leading market for global fast food, restaurant, and hotel and hotel brands. Moreover, the country has a resilient currency and effective monetary policy. However, with the recent incidences of global financial crises, the US economy has become unstable following an increasing level of monetary policy uncertainty. Considering that businesses and hospitality-related activities may be susceptible to financial distortions, this study extends the literature by examining not only the asymmetric effect of exchange rate and monetary policy uncertainty on RHP but also by identifying an asymmetric causality between these variables over the period 2001:M12 to 2019:M01. Empirical results from the NARDL provide evidence of asymmetry concerning the direction of exchange rate, monetary policy uncertainty, and energy price shocks. Furthermore, we found that a positive exchange rate shock (appreciation) causes RHP to fall, but a negative exchange rate shock (depreciation) of the same magnitude causes RHP to increase. Moreover, a positive shock in monetary policy uncertainty decreases the prices of restaurants and hotels, while a negative shock of identical size increases the prices of restaurants and hotels in the US. A close examination of the findings indicates that both negative exchange rate and negative monetary policy uncertainty shocks (depreciation) have stronger impact on RHP. Moreover, a positive energy price shock increases RHP, but a negative shock of the same magnitude has no significant impact on RHP both in the long and short run.

Furthermore, results of the asymmetric causality indicate that a positive shock in the exchange rate causes a positive shock to RHP. Positive and negative shocks in monetary policy uncertainty have predictive power for positive and negative shocks in the exchange rate and vice versa. This suggests asymmetric feedback effect between monetary policy uncertainty and exchange rate. Moreover, asymmetric causality is detected moving only from a positive (negative) shock in the exchange rate to a positive (negative) shock in energy price.

Therefore, these findings contain policy implications for stabilizing the US economy and achieving low and stable price levels. The findings provide insights for policymakers to attain price stability in the US hospitality-related industries. Particularly, our findings would provide insights to policymakers to help design appropriate monetary policies against domestic and global shocks. Recently, the US dollar and major exchange rates worldwide have experienced sharp responses to issues associated with fiscal policy arising from the political polarization on contentious issues of debt ceiling and other fiscal policy dichotomies.

Finally, our analysis contains some limitations. Our analysis excludes the COVID-19 pandemic period owing to data unavailability. Hence, we recommend that future studies conduct a similar investigation while extending the investigation period to accommodate the coronavirus pandemic period. Moreover, such studies can capture the effect of COVID-19 in the pass-through channels. Researchers can consider a panel study that includes tourist destinations that severely affected the COVID-19 pandemic (e.g., the US, Spain, Italy, Brazil, and others) in the future.

## Data Availability

Not applicable.

## Abbreviations

ADF: Augmented Dickey-Fuller
ARDL: Autoregressive distributed lag
BDS: Broock, Scheinkman and Dechert
COVID-19: Coronavirus
CPI: Consumer price index
ECT: Error correction term
EPR: Price of energy
EPU: Economic policy uncertainty
HJC: Hatemi-J criterion
IID: Independently and identically distributed
KPSS: Kwiatkowski-Phillips-Schmidt-Shin
LN: Logarithmic function
LSGDM: Large-scale group decision-making
L-S: Lee-Strazicich
MP: Monetary policy uncertainty
NARDL: Non-linear autoregressive distributed lag
NEER: Nominal effective exchange rate
PP: Phillips-Perron
PPP: Purchasing power parity
RESET: Regression equation specification error test
RHP: Restaurant and hotel prices
US: United States
VAR: Vector Autoregression

## Footnotes

1 Theoretically and empirically, energy prices are one of the major determinants of prices (see Delatte & Lopez-Villavicencio 2012).

2 Where subscript ‘*i*’ represents the variables we explored in this study, *i* = 1, 2, 3.

3 Crude oil dominates the energy market. Whatever happens to oil prices affects all other energy commodities (See Balcilar et al. 2022).
